# Dynamic transcriptomic analysis of the early response of female flowers of *Populus alba* × *P*. *glandulosa* to pollination

**DOI:** 10.1038/s41598-017-06255-3

**Published:** 2017-07-20

**Authors:** Pian Rao, Zhong Chen, Xiaoyu Yang, Kai Gao, Xiong Yang, Tianyun Zhao, Siyan Li, Bo Wu, Xinmin An

**Affiliations:** 1 National Engineering Laboratory for Tree Breeding, Key Laboratory of Genetics and Breeding in Forest Trees and Ornamental Plants of the Ministry of Education, College of Biological Sciences and Biotechnology, Beijing Forestry University, Beijing, 100083 China; 2 Key Laboratory of Silviculture and Conservation of the Ministry of Education, College of Forestry, Beijing Forestry University, Beijing, 100083 China; 3 Department of Forest Ecosystems and Society, Oregon State University, Corvallis, OR 97331 USA

## Abstract

Pollination is an important event in plant sexual reproduction, and post-pollination response is an essential process for reproduction. *Populus alba* × *P*. *glandulosa* is used widely in scientific research, especially in cross breeding as parents. Adult female *P*. *alba* × *P*. *glandulosa* flowers are highly compatible with pollen from male *P*. *tomentosa*, but the early post-pollination response of flowers at the molecular levels is unclear. In this study, RNA-seq was employed to comprehensively understand the response of female *P*. *alba* × *P*. *glandulosa* flowers to pollination. Enrichment analysis reveals that the ‘plant hormone signal transduction’ pathway is enhanced during pollen-pistil interaction. Moreover, genes related to auxin, gibberellin and ethylene biosynthesis were significantly up-regulated. Ca^2+^ and H^+^-related genes and cell wall-related genes are interrelated, and all of them are essential for pollen tube elongation in pistil, especially, free Ca^2+^ providing a concentration gradient for pollen tube guidance and involved in signal transduction. Furthermore, RNA-seq results indicate that genes involved in the adhesion and guidance for pollen germination and pollen tube growth are abundantly present in the extracellular matrix. Our study provides an overview and detailed information for understanding the molecular mechanism of early post-pollination response in this hybrid poplar reproduction.

## Introduction

Pollination is an essential process for reproduction and generation of offspring in plants. Pollens germinate on the surface of the stigma, and the sperm cells are delivered to the ovule through the pollen tube. During this process, the primary pollination signals from the pollens are received by the stigma during fertilization. The physiological and biochemical characteristics and morphological structures of female floral organs create certain changes to attract the pollen tube to penetrate the stylar tissue and enter the ovary. During this period, more signals are received and new responses are made in distal floral organs^1^.

The results of multiple experiments show that hormones are involved in signal transduction in response to pollination in plants. The pollen, during germinating on the stigma, will induce the synthesis of endogenous ethylene in floral organs, which acts as a signal molecule and is involved in a large number of subsequent developments in female flowers after pollination^1, 2^. 1-Aminocyclopropane-1-carboxylic acid (ACC) synthase and ACC oxidase take part in the catalytic process for the conversion of S-adenosylmethionine to ACC and then to ethylene. These expressions will significantly increase in post-pollinated flowers. In addition to the effect of ethylene on pollen tube growth, the internal organs will also secrete pollen tube attractants to direct the growth of pollen tube towards ovules, resulting in greater i ntercellular communication^3, 4^. The ovary starts to grow under the stimulation resulting from pollination, and massive structural and active substances are synthesized for this development, which requires a remarkable amount of energy. Moreover, floral organ senescence and pigmental changes have been found in floral development after pollination.

Hybrid poplar, *Populus alba* × *P*. *glandulosa*, was artificially bred by the Forest Genetics Research Institute of Korea in 1956^5^ using *P*. *alba* and *P*. *glandulosa* as parental species. Its female parent, *P*. *alba*, is planted in many countries with strong resistance to drought and good timber, but the growth is slower than other white poplars and the rooting rate of green-wood cutting is low. *P*. *glandulosa* is male parent of *P*. *alba* × *P*. *glandulosa*, with fast growth and high cutting survival rate. Superior hybrid progenies are selected from all progenies after hybridization of female *P*. *alba* and male *P*. *glandulosa* and these *P*. *alba* × *P*. *glandulosa* with advantageous traits inherited from parents are widely applicable to greening and lumber production. Since this hybrid poplar has been introduced to China, it’s always used in poplar cross breeding as parents. Female *P*. *alba* × *P*. *glandulosa* is highly compatible with pollen from other poplars and pollen of male *P*. *alba* × *P*. *glandulosa* can be accepted by pistil of many poplars. Whether hybrid poplar is used as female or male parent, the seed number per inflorescence is more than some other poplars’ cross combination^6^, and those seed germination rate is high. Studies on artificial hybridization of female *P*. *alba* × *P*. *glandulosa* and male *P*. *abla* × *P*. *glandulosa* also were carried out, but the seed setting rates of different cross combinations (parents came from different clones) were different, and traits segregation happened in those progenies^7^. Meanwhile, Shufang, *et al*.^8^ found that seed setting rate of hybridization of female *P*. *alba* and male *P*. *alba* × *P*. *glandulosa* was 80% and seed germination rate was 75.32%, and transplanting survival rate of seedlings was 57.3% that traits segregation also happened in progenies.The clonal propagation is always used to maintain *P*. *alba* × *P*. *glandulosa* traits, and progeny superior clones will be selected in interspecific cross breeding. Until now, studies on poplar reproduction are mainly focused on floral bud development and morphological changes. Researches on the response of female poplar flowers to pollination and flower development at molecular level are rare. The mobilization of self-genes to adjust its mRNA levels and change the internal and external environment of cells, to promote pollen tube growth in pistil and stimulate ovary development, in a relatively short time after pollination is unknown. Therefore, this study is focused on the process of reproduction in female flowers of this hybrid poplar using RNA-seq technology to detect plant transcriptome, which can results in a comprehensive understanding to the molecular mechanism of early post-pollination response especially during crossing breeding using this hybrid poplar as female parent.

## Results

### Morphological changes of pistil and pollen tube

**Figure 1:**
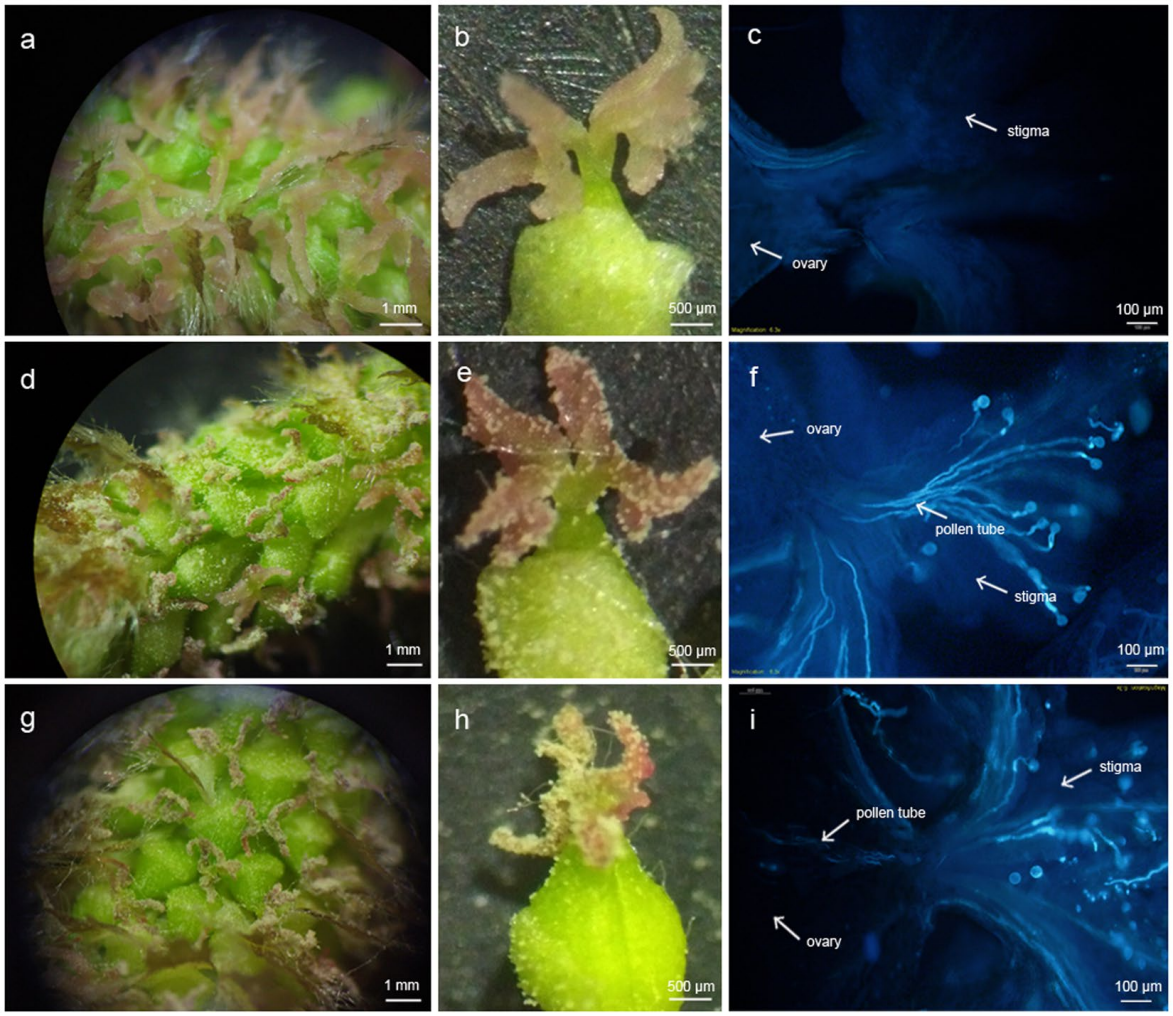
Morphological changes of pistil and pollen tube. (**a,b** and **c**) Represent observations made in samples collected at 0 h. Female flowers in middle positon of catkin were in the best pollination period and stigma crack angle was about 180°, but no pollen germination on surface of stigma. (**d**,**e** and **f**) Were samples obtained at 12 h, and a large number of pollen germinated and pollen tube grew in style. (**g,h** and **i**) Were collected at 24 h. Stigma senescence occurred and the tip of pollen tube had entered in ovary.

The female inflorescence was completely exposed and the stigma of the female flower extended when the *P*. *alba* × *P*. *glandulosa* branches were cultured in water at greenhouse for 7–10 days. When the stigma crack angle is about 180°, it is receptive period for pollination (Fig. 1a,b). The stigma of *P*. *alba* × *P*. *glandulosa* female flower is fresh and crystal clear during the best pollination period, but after pollination, it turns dark and wilting (Fig. 1d,e,g and h). The result of aniline blue staining shows that at 12 h, the pollen tube grows through the stylar tissue (Fig. 1f). At 24 h, pollen tube tip has enter into the ovary (Fig. 1i).

### Deep sequencing, *de novo* assembly, and function annotation

**Figure 2:**
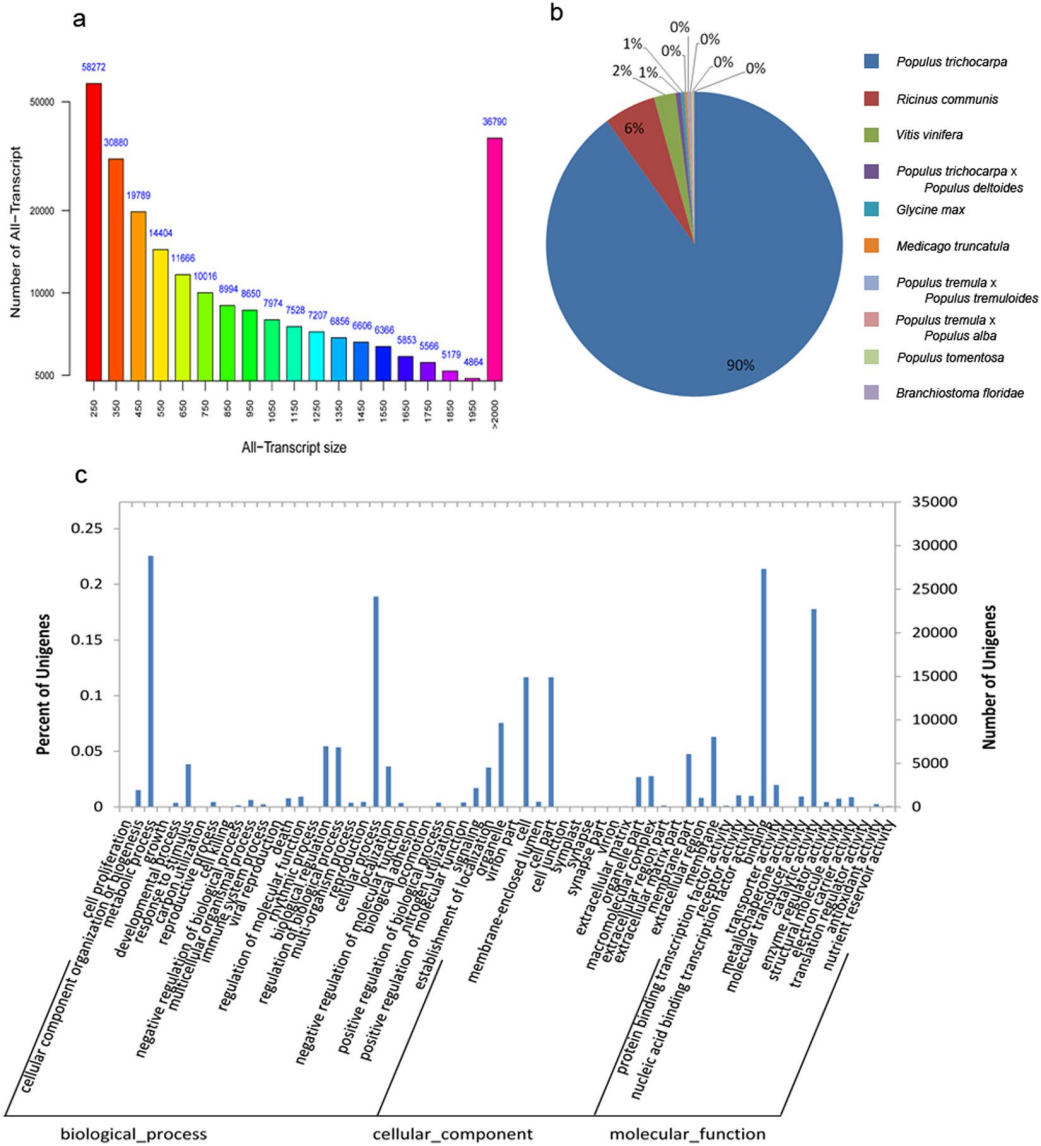
Characteristics of samples UniGenes. (**a**) Length distribution of all-unigene, (**b**) percentage of unigenes matching the top 10 species using BLASTx in the NR database, (**c**) classification of GO annotations at the secondary level.

Three cDNA libraries were constructed for deep sequencing. A total of 132 million reads with an average length 94.6 bp were available and the valid ratio was 83.70% (Table S1). Approximately 500,000 reads were selected randomly from post-quality control date to BLAST against nucleotide database for empoison detection. The results showed that the top 10 species with the greatest similarity were *Populus*, except the 10^th^ species, *Picea glauca* (Table S2). All valid reads from their samples were assembled to generate 263,460 transcripts with length greater than 200 bp and 127,811 unigenes with N50 values of 1,665 bp and 1,310 bp for transcripts and unigenes, respectively (Table 1). The length and distribution of all assembled unigenes were presented in Fig. 2a.

**Table 1:** The result of *de novo* assembly.

	All (>=200 bp)	>=500 bp	>=1000 bp	N50	N90	Total Length	Max Length	Min Length	Average Length
Transcript	263460	154680	100882	1665	419	267095067	10474	201	1013.8
Unigene	127811	47566	26911	1310	277	90881861	10474	201	711.06

Unigenes from all samples were annotated by BLASTx to many public database, including nucleotide database from the National Center for Biotechnology Information (NCBI) non-redundant (NR) database and conserved domain database (CDD), with the similarity greater than 0.3 and E-value threshold of 1e^−5^. Approximately 45.14% (57,688) unigenes could be annotated to NR database. Based on NR annotation, 97.11% unigene sequences can be mapped with sequences from 10 top species (Fig. 2b). Approximately 90% of these unigenes had greatest similarity to *P*. *trichocarpa*, while the remaining unigenes had greater similarity to *Ricinus communis* (6%) and *Vitis vinifera* (2%). Furthermore, 22.61% (28,892), 45.52% (58,178), 19.97% (25,518), and 36.94% (47,210) of sequences shared a greater similarity to proteins in SWISS-PROT, TrEMBL, CDD, and Pfam databases, respectively. In addition, 15,584 unigenes were annotated to Clusters of orthologous groups for functional categories of eukaryotic complete genomes(COGs) database. The results demonstrated that these sequences were annotated to 25 COGs functional categories (Figure S1). The three top categories with most annotated unigene sequences were ‘signal transduction mechanisms’ (T; 3,357 unigenes), ‘posttranslational modification, protein turnover, chaperones’ (O; 2,704 unigenes), and ‘general function prediction only’ (R; 2,479 unigenes).

After the Kyoto Encyclopedia of Genes and Genomes (KEGG) analysis, 16,104 unigenes were mapped to 978 enzymes and 295 KEGG pathways. The comparison of all unigenes with the KEGG database showed that the pathways with highest representation were ‘ribosome’ (ko03010, 610 unigenes), ‘plant hormone signal transduction’ (ko04075, 475 unigenes), ‘protein processing in endoplasmic reticulum’ (ko04141, 460 unigenes), ‘starch and sucrose metabolism’ (ko00500, 441 unigenes), and ‘spliceosome’ (ko03040, 397 unigenes) (Table S4). The gene ontology(GO) classification was implemented, and 41,451 unigenes were annotated to 64 GO termsand these sequences distribution was shown in Fig. 2c.

### Differentially expressed transcriptsin post-pollinated flowers

Two sets of differentially expressed genes (DEGs) between two adjacent time samples were analysed respectively. Approximately 62,248 unigenes (32,327 up-regulated unigenes and 29,921 down-regulated unigenes) showed differential expression between 0 h and 12 h, and 53,717 DEGs (27,888 up-regulated unigenes and 25,824 down-regulated unigenes) were identified between 12 h and 24 h based on fold change (Table S3). In addition to the top three KEGG pathways, including ‘cell cycle,’ ‘starch and sucrose metabolism,’ and ‘phenylpropanoid biosynthesis’, there were many differentially expressed transcripts (DETs) significantly enriched to ‘plant hormone signal’ transduction’ pathway, which is related to the growth of pollen tube, with p-value threshold of 0.05. The GO enrichment analysis revealed that 19,273 and 15,278 DETS were significantly enriched from two sets of continuous time samples, respectively, with a criterion of p < 0.05. The major GO terms with highest DGTs, including ‘ATP binding’, ‘nucleotide binding’, ‘membrane’, ‘integral to membrane’, ‘DNA binding’, ‘oxidation-reduction process’ and ‘oxidoreductase activity’ were enriched in all samples after pollination. Moreover, the enrichment of some GO terms that were essential for pollen tube elongation and post-pollinated flowers development, including ‘pectinesterase activity’, ‘pectate lyase activity’, ‘ethylene biosynthetic process’, ‘embryo development’, and ‘auxin-mediated signaling pathway’ were also significant.

### Hormone-related genes

**Figure 3:**
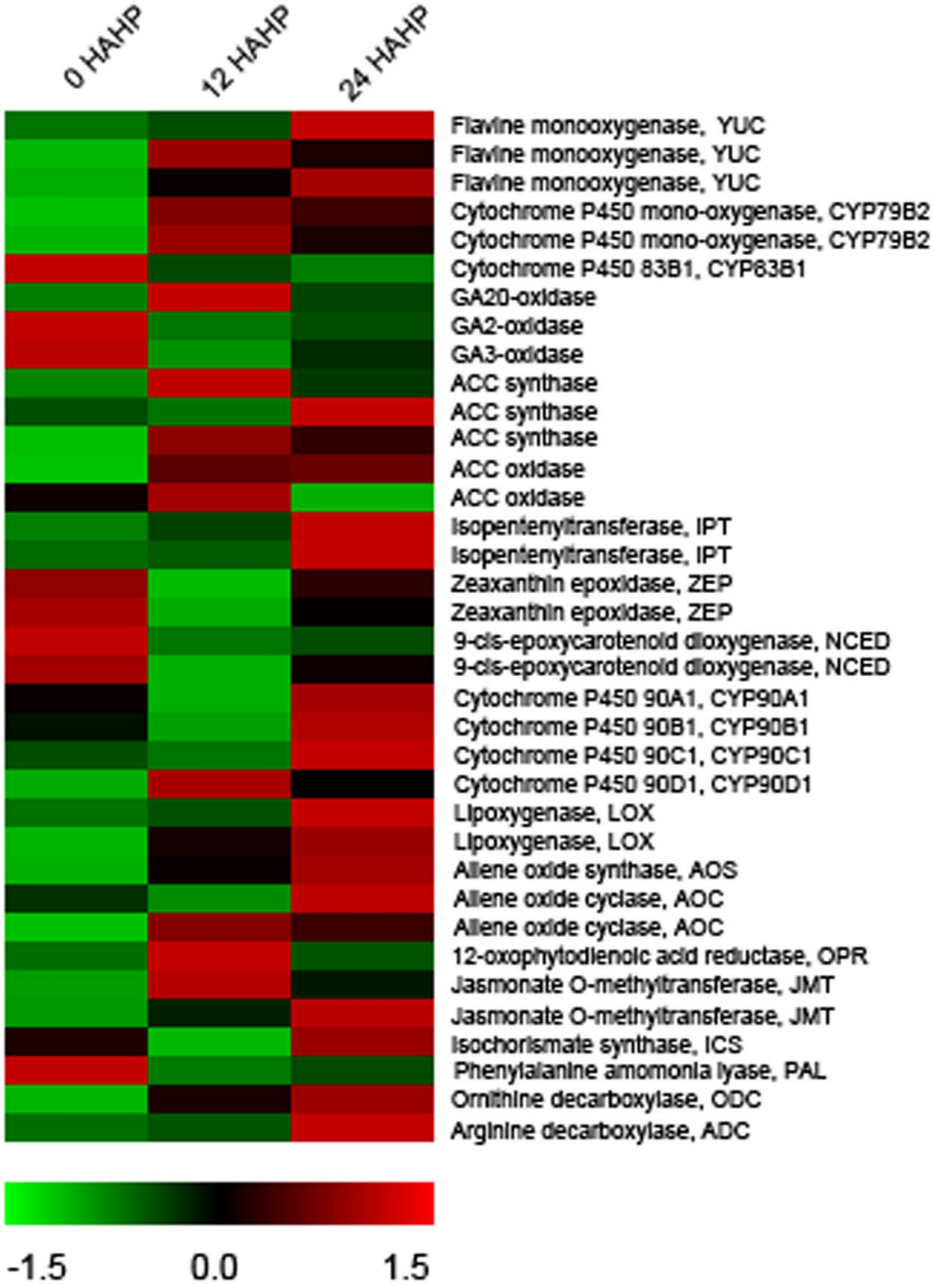
Genes involved in plant hormones biosynthesis.

Differential expression of hormone-related genes was analysed, and those DETs related to hormone synthesis, response factor, and transportation were enriched. YUCCA flavine monooxygenase (YUC) and cytochrome P450 monooxygenase CYP79B2 were involved in two different auxin biosynthesis pathways, respectively. Those genes showed elevated expression at 12 h (Fig. 3). Cytochrome P450 83B1 (CYP83B1) catalysed the indole-3-acetaldoxime, a precursor for auxin biosynthesis, into indole-3-S-alkyl-thiohydroximate that can result in precursor shortage for auxin. Essentially, we detected that mRNA level of CYP83B1 was significantly down-regulated after pollination (Fig. 3). Auxin influx carrier, efflux carrier, and response-related genes were also identified. Gibberellins (GAs) biosynthesis-related genes were examined, for example, the expression of GA20-oxidase genes exhibited a significant up-regulation, but a slight down-regulated expression of GA3-oxidase gene was also detected (Fig. 3). The significant up-regulated expression of ACC synthase involved in ‘ethylene biosynthesis process’ was detected at 12 h, but ACC oxidase exhibited a relatively unchanged expression with very slight elevation (Fig. 3). Additionally, those genes belonging to ERF gene family and encoding ethylene response factors were also analysed.

Cytokinin (CTK) and abscisic acid (ABA)-related GO terms also were identified, and the expression of genes related to these hormones was examined (Fig. 3). Cytokinin biosynthetic isopentenyltransferase (IPT), a rate-limiting enzyme of cytokinin biosynthesis in plants, exhibited a higher abundant expression after pollination. Zeaxanthin epoxidase (ZEP) and 9-cis-epoxycarotenoid dioxygenase (NCED) are involved in abscisic acid biosynthesis, and NCED is an important rate-limiting enzyme for abscisic acid biosynthesis process. These genes of ZEP exhibited a trend in which the expression first decreased and then increased again. NCED genes shared a similar trend with ZEP, but their expression had a smaller margin rise from 12 hours to 24 hours.

In addition to these five hormones, a significant enrichment of brassinosteroid (BR), jasmonate (JAs), salicylic acid (SA), and polyamine-related GO terms was also identified. CPD/CYP90A1, DWF4/CYP90B1, and ROT3/CYP90C1 are key genes in BR biosynthesis process. No significant changes in CYP90A1, CYP90B1, and CYP90C1 were detected, but we detected a slight up-regulated expression between 12 and 24 h (Fig. 3). CYP90C1 and CYP90D1 might be redundant roles as BR C-23 hydroxylase^9^, and CYP90D1 exhibited an up-regulated expression between 0 and 12 h. The major genes involved in JAs biosynthesis include lipoxygenase (LOX), allene oxide synthase (AOS), allene oxide cyclase (AOC), and 12-oxophytodienoic acid reductase (OPR). In addition to OPR, the other genes basically exhibited an up-regulation and jasmonate O-methyltransferase (JMT), catalysing the biosynthesis of volatile methyl jasmonate, was also detected to be up-regulated (Fig. 3). Isochorismate synthase (ICS) and phenylalanine ammonia lyase (PAL) are important for SA biosynthesis, respectively. No significant changes of ICS, but a remarkable down-regulation of PAL was detected at 12 h (Fig. 3). Ornithine decarboxylase (ODC) and arginine decarboxylase (ADC) related to putrescine, the central product in synthesis pathway of polyamine, were examined and both of them exhibit an elevated expression (Fig. 3). Polyamine oxidase gene involved in polyamine catabolism was also identified.

### Validation by real-time quantitative PCR

**Figure 4:**
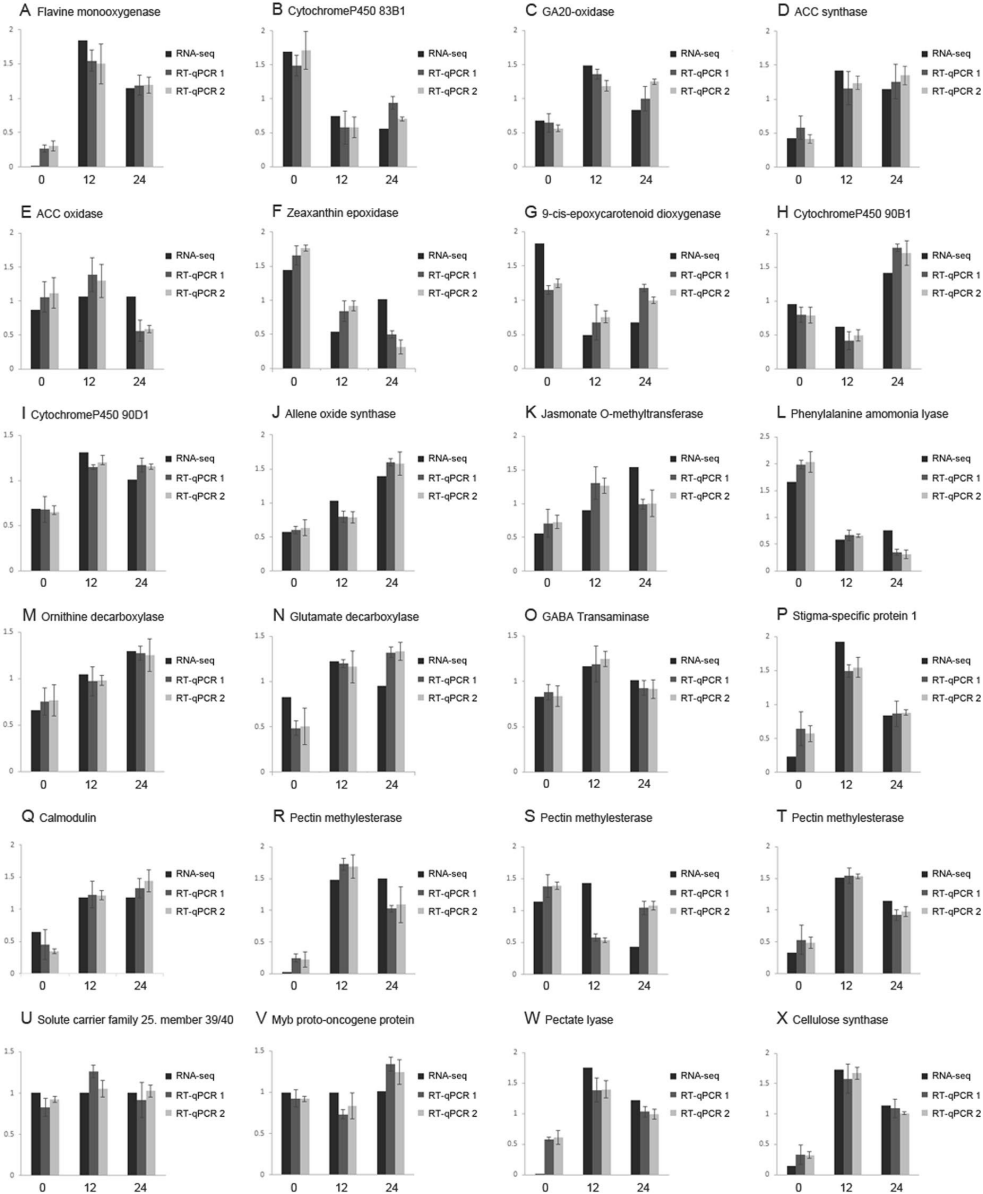
Comparison of transcript abundance determined by RNA-seq and RT-qPCR in 0, 12, 24 h samples. RNA-seq, results of transcript abundance determined by RNA-seq are shown with a black colar. RT-qPCR 1, results of transcript abundance determined by RT-qPCR using *Actin* gene as reference gene are shown with a gray colar, and RT-qPCR 2, results of transcript abundance determined by RT-qPCR using *Ubiquitin* gene as reference gene are shown with a lighter gray colar.

To validate the RNA-seq results of three samples from pollinated flowers in present study, we chose 24 genes, which were related tohormones biosynthesis, cell wall modification and subsequent breakdown, some ions, and guidance of pollen tube. The results of real-time quantitative PCR (RT-qPCR) were compared to gene expressions obtained by RNA-seq, and it indicated that the two sets of results on genes expressions obtained by the two methods were similar (Fig. 4).

## Discussion

The stigma of *P*. *alba* × *P*. *glandulosa* female flower looks fresh and crystal clear in optimum pollination period. However, stigma senescence occurs in pollinated flowers at 24 h, which can be visible through the darker colour of the wilting stigma (Fig. 1a,b,d,e,g and h). The aniline blue staining of pollinated flowers from the three samples reveals that the pollen germinates and the pollen tube grows in the stylar tissue at 12 hours after pollination. Later, the pollen tube begins to enter the ovary, and pollen tube tip grows at 24 h (Fig. 1c,f and i). According to previous studies, the time required for fertilization in *P*. *alba* × *P*. *glandulosa* female flowers is typically about 120 hours after pollination. Therefore, these changes during 24 h are only focused on the female response and its development stimulated by pollination rather than late development or seed formation.

### Association between post-pollination response and hormones

Plant hormones, also called phytohormones, are involved in a whole range of developmental process, including vegetative and reproductive growth, and its changes in very low concentrations will cause significant responses.

Till date, multiple plant hormones have been proved to be involved in post-pollination regulation as signal molecules^10^, auxin being one among them. Exogenous pollen-borne auxin is one of the primary pollen signals, which stimulates ethylene production in stigma and initiates the ovary development after pollination^1^. Application of auxin antagonist or auxin transport inhibitor before pollination, resulting in a nullified pollinated-induced ovary development, also suggested that auxin regulates ovary development^11–15^. Apart from ovary development, auxin also stimulates pollen germination and growth of the pollen tube. Wu, *et al*.^16^ proved that auxin could promote pollen germination and pollen tube growth *in vitro*, and these hormones levels were elevated in the stigma of pollinated torenia (*Torenia fournieri*) plants. Similarly, in our study, the genes encoding YUC and CYP79B2 exhibited an up-regulation in post-pollinated flowers (Fig. 3). Additionally, CYP83B1 showed a significant down-regulated expression. The result indicated that the concentration of endogenous auxin might increase after pollination. It is obvious that endogenous auxin is also essential for ovary development and pollen tube growth *in vivo* in the present study.

Similar to auxin, GAs also control plant reproductive development. The increase in GAs concentrations will be induced by pollination in many plants, and auxin can promote GAs biosynthesis^11, 17, 18^. Serrani, *et al*.^18^ indicated that the activation of GAs biosynthesis is due to the up-regulation of GA20-oxidase initiated by pollination in tomato (*Solanum lycopersium*). The effect of GAs in parthenocarpy was visualized through GAs application in unpollinated flowers and emergence of tomato mutant plants, including *pat*-2 and *pat*-3/*pat*-4^14, 19, 20^. GAs are required for pollen tube growth and will stimulate pollen tube elongation *in vitro* ^16, 21^. GA20-oxidase exhibited a significant up-regulated expression in our pollinated poplar flowers, which was similar to related studies (Fig. 3). This result indicates that GA biosynthesis is also activated by pollination, and the GAs are involved in the growth of pollen tube and ovary development in poplar reproduction.

Ethylene biosynthesis of pistil was triggered by pollen-borne auxin, and the initiation in stigma also resulted from pollen-borne ACC, which were both beneficial for endogenous ethylene production due to autocatalysis^1^. The regulation of endogenous ethylene biosynthesis occurs in many plants in response to pollination^22–24^. Previous studies have proved that ethylene is essential for pollen germination and pollen tube growth in pollinated flowers^2^, and the elongation of pollen tube depended on an optimum elevated ethylene level. In our pollinated poplar flowers, the expression of ACC synthase was up-regulated (Fig. 3), which might indicate that ethylene biosynthesis was enhanced after pollination. It is possible that ethylene production has a similar function in pollen tube growth in poplar flowers. Pollination will induce the corolla and transmitting tract(TT) senescence^1, 22, 25, 26^, but female poplar flowers have only the bracts and pistils without petals. Therefore, it is the senescence of stigma and stylar tissue induced by pollination that we may visually notice. Hormones, especially ethylene and ABA, are closely related to senescence^27–29^. In the present study, the up-regulation of IPT suggested that CTK biosynthesis is enhanced, and the elevated CTK concentration can result in delaying senescence. ABA production will be delayed by overexpression of CTK and its concentration is also affected by changes in ethylene concentration, which is also related to senescence^29^. Our experiment indicates that NCED and ZEP expression decrease in the first period (from 0 to 12 h) and increase in second period (from 12 to 24 h) (Fig. 3), resulting in a rise after the initial drop in ABA concentration. CTK also induces parthenocarpy by enhancing auxin and GAs biosynthesis in tomato^30^, but ABA will inhibit pollen tube growth *in vitro* and the ABA contents of stigma will decrease in pollinated *T*. *fournieri* L.^16^.

In addition to those five hormones, others also are related to post-pollination response and development. BR and polyamine will sitimulate pollen tube growth^31–34^, and Jas and SA are also related to fruit development and senecense. Therefore, how phytohormones ensure the balance of pollen tube growth, ovary development and TT senescence is worth further studing and discussing.

### Ca^2+^ supply

**Figure 5:**
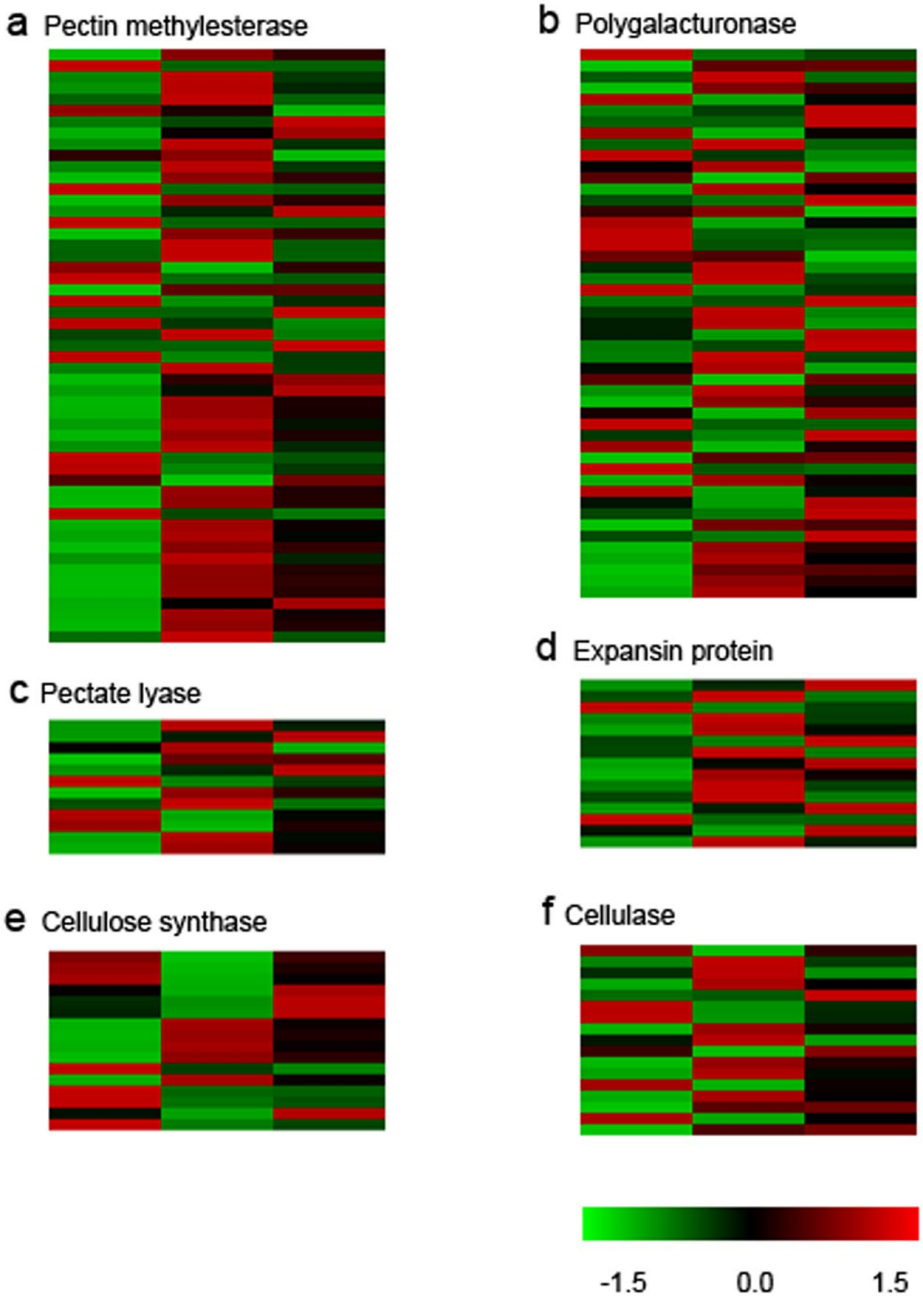
Heat map of cell-wall related genes expression. (**a**) Genes encoding pectin methylesterase, (**b**) genes encoding polygalacturonase, (**c**) genes encoding pectate lyase, (**d**) genes encoding expansin protein, (**e**) genes encoding cellulose synthase, (**f**) genes encoding cellulase.

Pectin is one component of cell wall and pollination that can trigger pectin methylesterase (PEM) synthesis and loosen cell wall of stylar TT.In our pollinated flowers, the genes expression of PEM is significantly elevated (Fig. 5a). The PEM catalyses esterified pectin into unesterified pectin. But deesterification process will result in the rise of unesterified pectin, which can combine with divalent calcium ion to form a more stable structure, the Ca^2+^-binding pectin^35^. This group of unesterified pectins need to be hydrolysed further to loosen the cell wall. Unesterified pectins are a source of Ca^2+^ for pollen tube growth. The lysis of Ca^2+^-binding pectins by cell wall-loosening hydrolases, including polygalacturonases and pectate lyases, will produce loosely bound Ca^2+^, which can form an essential Ca^2+^ gradient. The increase in loosely bound Ca^2+^levels induced by pollination and pollen tube growth has been proved in many plants^36^. The degradation of Ca^2+^-binding pectin creates optimum calcium medium for pollen tube growth and guidance.

In present study, the mRNA levels of polygalacturonases and pectate lyases genes mostly increase following pollination (Fig. 5b,c). It suggests that more hydrolases participate in cell wall loosening. However, the action of these cell wall-loosening hydrolases must be promoted by the reduction of pH. Protons are released during the demethylesterification of homogalacturonan (esterified pectin) and result in the reduction of pH^35^. Therefore, the release of calcium ions is consequent and cell wall of stylar tissue can be loosened. These two results are beneficial for the pollen tube and its growth.

**Figure 6:**
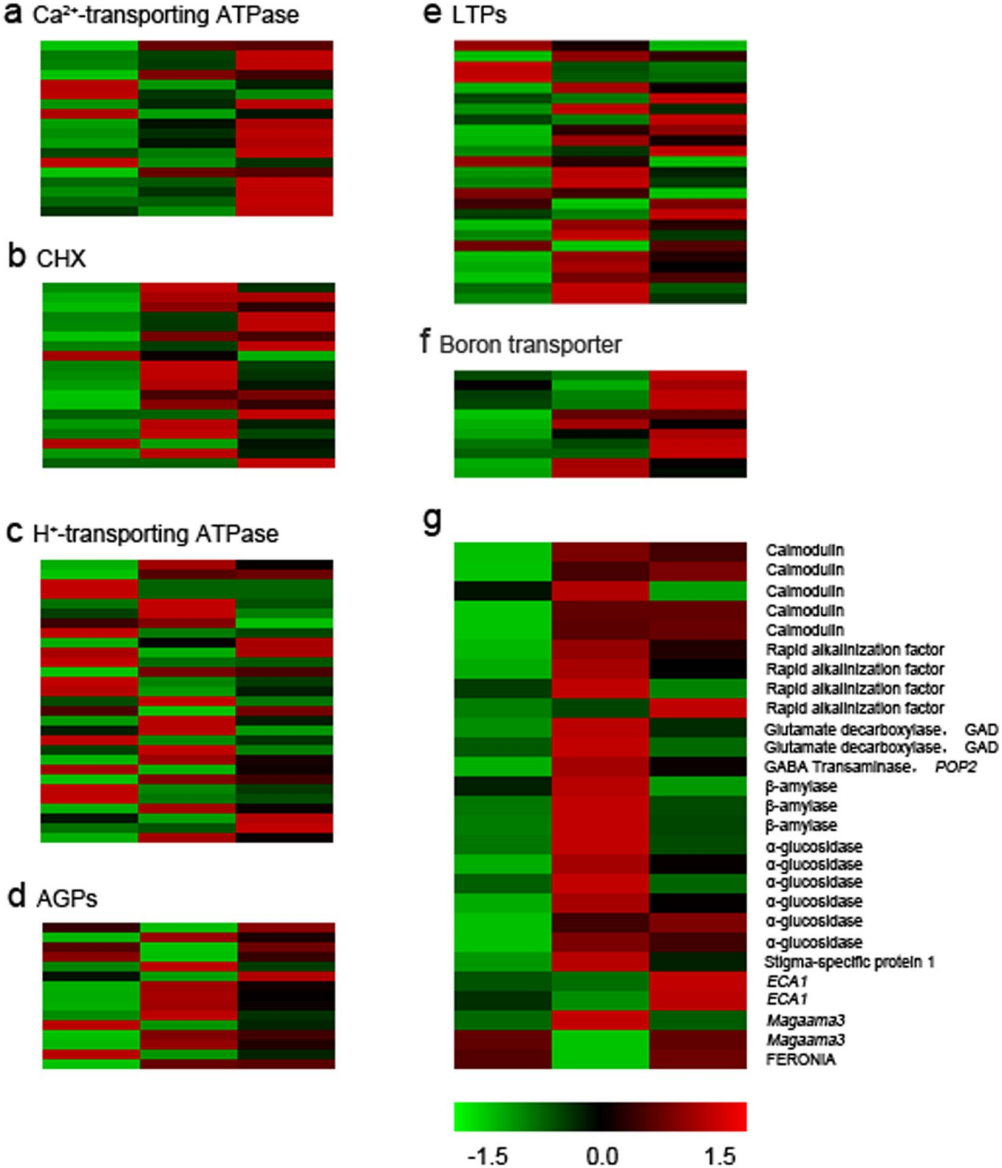
Genes related to pollen tube guidence. (**a**) Genes encoding Ca^2+^-transporting ATPase, (**b**) genes encoding CHX, (**c**) genes encoding H^+^-transporting ATPase, (**d**) genes encoding AGPs, (**e**) genes encoding LTPs, (**f**) boron transporter, (**g**) other related genes.

The maintenance of Ca^2+^ gradient also depends on Ca^2+^ pump gene that encodes Ca^2+^-transporting ATPase. These Ca^2+^-ATPases are involved in calcium transportation; for instance, auto-inhibited Ca^2+^-ATPase 13 (ACA13) in papilla cell of Arabidopsis stigma will promote Ca^2+^ export and successful fertilization^3^. The expression state of the Ca^2+^ pump genes in our pollinated poplar flowers also increased and some up-regulation of them were notable (Fig. 6a). This result indicates that the movement of calcium ion is very active during post-pollination regulation.

In addition to the polar growth of the pollen tube directly due to Ca^2+^ gradient, the calcium ion is also essential for signal transduction^37^. The expression state of related protein calmodulin (CALM) genes also increased. Obviously, signal transduction pathway, which requires calcium ions, may be enhanced during post-pollination development. Nitric oxide (NO) plays an important role in pollen tube growth and NO signalling can target the pollen tube to the ovule^38^. This pathway is also involved in Ca^2+^ signal^39^. Calcium ions are also related to FERONIA (FER) signal pathway^40^, which also is involved in pollen tube guidance due to FER, a synergid-expressed kinase.

A reduction of wall pH is also related to the action of plasma membrane H^+^-ATPase. The proton is pumped by H^+^-ATPase to the cell wall, which is beneficial for acid-induced growth. Additionally, cation proton exchanger (CHX) is also responsible for proton transportation and can regulate pH in plant cells. Certainly, H^+^-ATPase and CHX are also related to translocation of metal ions, including Ca^2+^. In the present study, most transcription of H^+^-ATPase and CHX are up-regulated (Fig. 6b,c). Particularly, the mRNA level of CHX genes was significantly elevated at 12 h, with several down-regulated genes expression.

### Cell wall loosening

After pollen germination on the stigma surface, the process of pollen tube elongation in the extracellular matrix (ECM) of the pistil tissue is essential for transfer of male gametes into the blastocyst. Cell wall loosening of stylar tissue will be beneficial for pollen tube growth. Therefore, many genes related to cell wall modification and breakdown exhibited significant changes in expression in pollinated flowers, which results not just from ovary development stimulated by pollination.

The action of PEM will cause cell wall loosening with changes of calcium ions and pH. Polygalacturonases and pectate lyases also participate in this process. Cellulose is another component of plant cell wall. During the process of ovary development after pollination, cellulose synthesis and degradation are essential for cell enlargement and division. In the present study, cellulose synthases, which are involved in cellulose production, state increased especially at 24 h (Fig. 5e), which suggest that cellulose synthesis is enhanced after pollination, particularly at 24 h when the ovary is significantly enlarged. Celluloses involved in degradation of cellulose also exhibit a degree of change (Fig. 5f). Besides, DETs of cell wall-related genes, including xylanase, chitinase, and callose synthase genes, have been detected. These enzymes more or less refer to post-pollination regulation of the growth of the ovary.

Expansin superfamily proteins are not enzymes with catalytic activity but have cell wall-loosening properties^41, 42^. Under the acidic optimum regulated by plasma membrane H^+^-ATPase, the enlargement of plant cell is remarkable^41, 42^. During this nonenzymatic mechanism, noncovalent bond between matrix polysaccharides and cellulose microfibrils, or each other, was disrupted by expansin, which acts like a zipper to move microfibrils apart from each other^42^. In addition to expensin proteins in pollen tube, some pistil-specific extensin-like proteins in stylar TT can also contribute to pollen tube growth or softening the cell wall of stigma to facilitate the penetration of pollen tube through the stigma and stylar tissues^41, 43–45^. In the present paper, expansin proteins show an abundant expression (Fig. 5d), suggesting that pollination will also induce these proteins to accelerate cell wall loosening of stylar tissue in this poplar flowers.

### Subtances in the ECM of female tissue

Transmitting tract-specific glycoprotein, an arabinogalactan protein (AGP), is abundant in the styles’ ECM of tobacco and chemotropically active for pollen tube growth^46^. The function of AGP during reproduction, especially cell adhesion and guidance for the orientated growth of pollen tube, has been confirmed^3, 46, 47^. The expression of most of the AGP genes increased in poplar flower following pollination (Fig. 6d), consistent with changes of AGPs in other plants. The transcript level of lipid transfer protein (LTP) genes has been detected in the present study (Fig. 6e). The LTPs belonging to cysteine-rich peptides (CRPs) have diversified functions in plant growth and reproduction. A class of stigma/style cysteine-rich adhesion-like LTP, secreted from pistil TT epidermis, is thought to be related to adhesion and mediated guidance of pollen tube growth when combined with pectin^46, 48–50^. The cell wall-loosening activity LTPs also have been proved^50^. Most of LTPs we detected exhibited a significantly elevated expression at 12 h. Obviously, the activity of LTPs on reproduction is enhanced at 12 h when the pollen tube grows in the style. Stigma-specific protein 1 (*STIG1)* gene is developmentally regulated and expressed specifically in the stigmatic secretory zone, which also belongs to CRPs and stimulates pollen tube growth in pistil^3, 49^. The up-regulated expression of *STIG1* (Fig. 6g) detected in the present study might also have a positive role in pollen germination and pollen tube elongation. But the lose of *STIG1* gene in petunia (*Petunia hybrida*) and tobacco (*Nicotiana tabacum*) does not affect pollen tube growth^51^, which indicates that this protein might be unnecessary.

γ-aminobutyric acid (GABA) is a small molecule, one of the substances in the ECM, that we analysed in this study. The role of GABA in reproduction is involved in stimulating pollen germination and pollen tube growth that might be realized via particular channel of the pollen tube^3, 48^. Glutamate decarboxylase (GAD) converts glutamate into GABA, and *pollen pistil interaction 2* (*POP2*), encoding a GABA transaminase, is involved in GABA degradation to form GABA gradient. Both GAD and *POP2* gene exhibited an up-regulation at 12 h (Fig. 6g). During the process of pollen tube growth in the style of poplar flowers, the elevated expression of genes at 12 h suggests that more GABA is synthesized and a putative GABA gradient can also be formed, which is similar to other plants. GABA level in *pop2* mutant style is higher than in wild-type style^48^. However, *POP2* in pollen is the true cause of GABA degradation to stimulate tip growth. The *pop2* mutant pollen without normal tube growth will result in fertilization failure, but wild-type pollen will grow normally in *pop2* style^48, 52^.

Sugar present in ECM, providing nutrition for pollen, is another important component in ECM^48^. Beta-glucosidase and beta-amylase involved in sucrose content changes are identified and those mRNA levels were up-regulated as we expected (Fig. 6g). This fact may suggest that the use of carbohydrates is enhanced to provide energy for pollen tube action in pistil or other activities in the pistil after pollination, especially between 0 and 12 h.

### Other essential genes for fertilization and dynamically expressed genes after pollination

Boron (B) plays an important role in plant growth, especially its reproductive growth. Under low B concentrations, plant reproduction organs will grow sluggishly; for example, B deficiency can affect pollen development and induce male sterility^53, 54^. An essential action is that B, at an appropriate concentration, will contribute to pollen germination and pollen tube elongation^55^. It also plays a major role in ovary development and pistil functions, and a high B concentration is generally present in female flower parts^53, 56^. We have detected a significantly elevated expression of B transporter genes at 12 h or 24 h (Fig. 6f). It is obvious that the utilization of B increases during post-pollination regulation.

The action of FER protein, a synergid-expressed receptor-like kinase, which was detected in present study (Fig. 6g). Those *fer* mutants will lose the capacity of arresting pollen tube growth and rupture of pollen tube tip to release the sperm cells in ovules, but pollen from *fer* mutants can achieve fertilization successfully in wild-type ovules^57^. Besides, RALF belonging to CPR family, can induce a signaling cascade as a ligand of FER in *A*. *thaliana* ^3, 4^. The RALF mRNA levels significantly increased after pollination in our study (Fig. 6g), which suggests that FER may work with RALF, but the realities about the action between FER and RALF are unknown in response to pollination and pollen tube growth. *Magatama3* (Fig. 6g) is another gene expressing in synergid cell and encoding a helicase, which is also essential for pollen tube guidance^58^. Two elevated mRNA levels of prolamin-like protein genes (Fig. 6g), which might belong to *ECA1* gametogenesis-related family with potential functions in lipid transfer or protection during plant embryo sac development and reproduction^49^, was detected, which exhibited a significant up-regulation between 12 and 24 h. After pollination, ovules will continue to develop in female flowers of *P*. *alba* × *P*. *glandulosa* for ovule maturation. These genes expressed in female gametophytes often regulate the development of female gametophytes or are involved in pollen tube guidance and their presence is essential even in the crux of fertilization.

## Conclusion

The changes of mRNA levels in this hybrid poplar flowers after pollination provide an overview and detailed information for understanding the molecular mechanism of early post-pollination response. During the response to pollination in poplar flowers, hormones are necessary. Those hormones will stimulate pollen germination and pollen tube growth, ovary development, fruit set, and even floral organ senescence. Meanwhile, the changes of biosynthesis of IAA, GAs, ethylene, CTK and ABA in pollinated hybrid poplars flowers are relatively conserved in some plants including torenia, tomato and tobacco, especially with similar functions on pollination response. Cell wall loosening facilitates pollen tube growth in TT of post-pollinated female flowers. The stylar of hybrid poplar female flower is not closed, and its pistil consists of stigma, ovary and hollow stylar with stylar canal pretty narrow and lined with rich-cytoplasm cells. Therefore, in different stylar, cell wall loosening to facilitate pollen tube growth might be relatively different. Ca^2+^ and other substances in ECM are essential for pollen tube growth, especially for adhesion and guidance. But researchers have found some genes are unnecessary, for example, the lose of *STIG1* gene in petunia and tobacco does not affect pollen tube growth. In summary, most genes during pollination response of poplar pollinated female flowers show similar expression trends with those in some other plants’ pollinated flower, but the differences still exist. Further studies are needed to elucidate the roles of these genes in the early post-pollination response of this hybrid poplar in the future.

## Methods

### Plant materials and sample collection

Branches were collected from three adult female *P*. *alba* × *P*. *glandulosa* trees belonging to same clone and one male *P*. *tomentosa* in February and then cultured in clean water in a greenhouse. The female flowers were pollinated with the pollen obtained from the branches of *P*. *tomentosa*, when the catkins of *P*. *alba* × *P*. *glandulosa* elongated and the flowers in middle position of inflorescence were under the optimal pollination period. These pollinated female flowers would be collectedat 0, 12, and 24 hours after hand-pollination respectively. Later, five pollinated catkins were collected from each female *P*. *alba* × *P*. *glandulosa* tree, and the flowers from the middle position of the catkins were pooled for isolating RNA at each sampling period.

### Morphological changes of samples

Pollinated flowers from the three samples were fixed in formalin-acetic acid-alcohol (FAA [5 mL formalin, 6 mL acetic acid, and 89 mL 50% ethanol]) for 24 h or subjected to long-term preservation as soon as they were taken from the branches. These FAA-fixed flowers were softened overnight with 8 M sodium hydroxide and gently rinsed thrice in a solution of potassium metaphosphate (4.17 mL 1 M dipotassium hydrogen phosphate, 0.83 mL 1 M potassium dihydrogen phosphate, and 995 mL deionized water at pH 7.5). The samples were immediately immersed in 0.5% water-aniline blue solution (formulated with 0.1 M potassium phosphate) for 1 h. After rinsing in the solution of potassium metaphosphate, the stained samples were observed under UV light using fluorescence microscope (BX61, Olympus, Tokyo, Japan).

### RNA extraction and deep sequencing

Total RNA was isolated separately from the samples of 0 h, 12 h, and 24 h by a modified method^59^ and treated with DNAse^59^. All three libraries were sequenced using an Illumina Hiseq 2000 platform according to paired-end sequencing. The raw reads were processed to remove low-quality read fragments by a slicing window method. The quality threshold was 20 bp and length threshold was 35 bp. Additionally, 500,000 reads were selected randomly from post-quality control date to BLAST against nucleotide database from the NCBI for empoison detection.

### *De novo* assembly and annotation of unigenes

*De novo* assembly of valid reads from three libraries was performed using the Trinity Assembly Program (version, trinityrnaseq_r2012-10-05) by paired-end assembly method. We selected the longest transcript at each locus as a unigene for the next analysis. Functional annotation of unigenes were performed by BLASTing against many protein databases, including NR database and CDD, with the similarity greater than 0.3 and E-value threshold of 1e^−5^. The KEGG analysis was achieved using the KEGG Automatic Annotation Server to obtain the corresponding KEGG pathway. Moreover, we counted the GO terms of three major categories, biological process, cellular component, and molecular function, respectively, based on the result of Blast-Uniprot.

### Expression analysis

All assembled transcripts were integrated to estimate the expression abundance. We used the mapping software bowtie 0.12.8 to map all valid reads back to transcriptome by single-end mapping method, and one read was allowed to map to multiple transcripts. A reads per kilobase of exon model per million mapped reads (RPKM) value was obtained. Among the three serial samples, two sets of DGEs between two adjacent-time samples must be analysed based on fold change in our study. The [|log_2_ (ratio)| ≥ 1] was set to search DETs. Hypergeometric distribution was applied to identify the significant GO terms and KEGG pathway with the whole transcriptome background. The enrichment analysis was performed at the significant level of 0.05 adjusted by false discovery rate.

### RT-qPCR

RT-qPCR was carried out on a 7500 Fast Real-time PCR System in a 50 μL reaction volume comprised of SYBR Premix EX Taq Kit (Takara, Kyoto, Japan), Primers, cDNA, and ddH_2_O, according the amplification protocol: 95 °C for 30 s, 95 °C for 5 s, 60 °C for 15 s, 72 °C for 20 s, for 40 cycles, and then 72 °C for 5 min. The cDNA was used as the template for PCR, which was synthesized by a reverse transcription system. We chose 24 genes to validate RNA-seq results, and the primers utilized are present in Table S5. *Actin* gene and *Ubiquitin* gene were used as a quantitative control^60, 61^.

## Electronic supplementary material

Supplementary Information
