# Supplementary material for: Dynamic transcriptomic analysis of the early response of female flowers of Populus alba × P. glandulosa to pollination
Source: Sci Rep. 2017 Jul 20;7:6048. doi: 10.1038/s41598-017-06255-3 (PMC5519698; doi:10.1038/s41598-017-06255-3)
Supplement: Supplementary file 1 — Supplementary Information [file 41598_2017_6255_MOESM1_ESM.pdf]

Supplementary Information

Dynamic transcriptomic analysis of the early response of female flowers of *Populus alba* × *P. glandulosa* to pollination

Pian Rao[1†], Zhong Chen[1,2†], Xiaoyu Yang[1], Kai Gao[1], Xiong Yang[1], Tianyun Zhao[1],Siyan Li[1], Bo Wu[1], Xinmin An[1,3*]

[1]National Engineering Laboratory for Tree Breeding, Key Laboratory of Genetics and Breeding in Forest Trees and Ornamental Plants of the Ministry of Education, College of Biological Sciences and Biotechnology, Beijing Forestry University, Beijing 100083, China

[2]Key Laboratory of Silviculture and Conservation of the Ministry of Education, College of Forestry, Beijing Forestry University, Beijing 100083, China

[3]Department of Forest Ecosystems and Society, Oregon State University, Corvallis, OR 97331, USA

[†]These authors contributed equally to this work.

* To whom correspondence should be addressed.

Tel/Fax: +86-010-62336248

E-mail: anxinmin@bjfu.edu.cn

Supplementary Information

Figure S1 COGs functional categories.

Table S1 Summary of sequencing and assembly results.

Table S2 The top ten species with the greatest similarity by selecting 500,000 reads randomly from post-quality control date to blast against nucleotide database for empoison detection.

Table S3 The number of differentially expressed unigenes base on different analysis method. FC.p: fold change AB positive, log2(B/A)>1, FC.n: fold change AB negative, log2(B/A)<-1, FT.g: fisher.testAB.greater, FT.l: fisher.testAB.less, CT: chisq.test.AB.

Table S4 The top ten pathways active in the transcriptome of all samples and the number of unigenes mapped to those pawthays.

Table S5 Primers utilized in RT-qPCR.

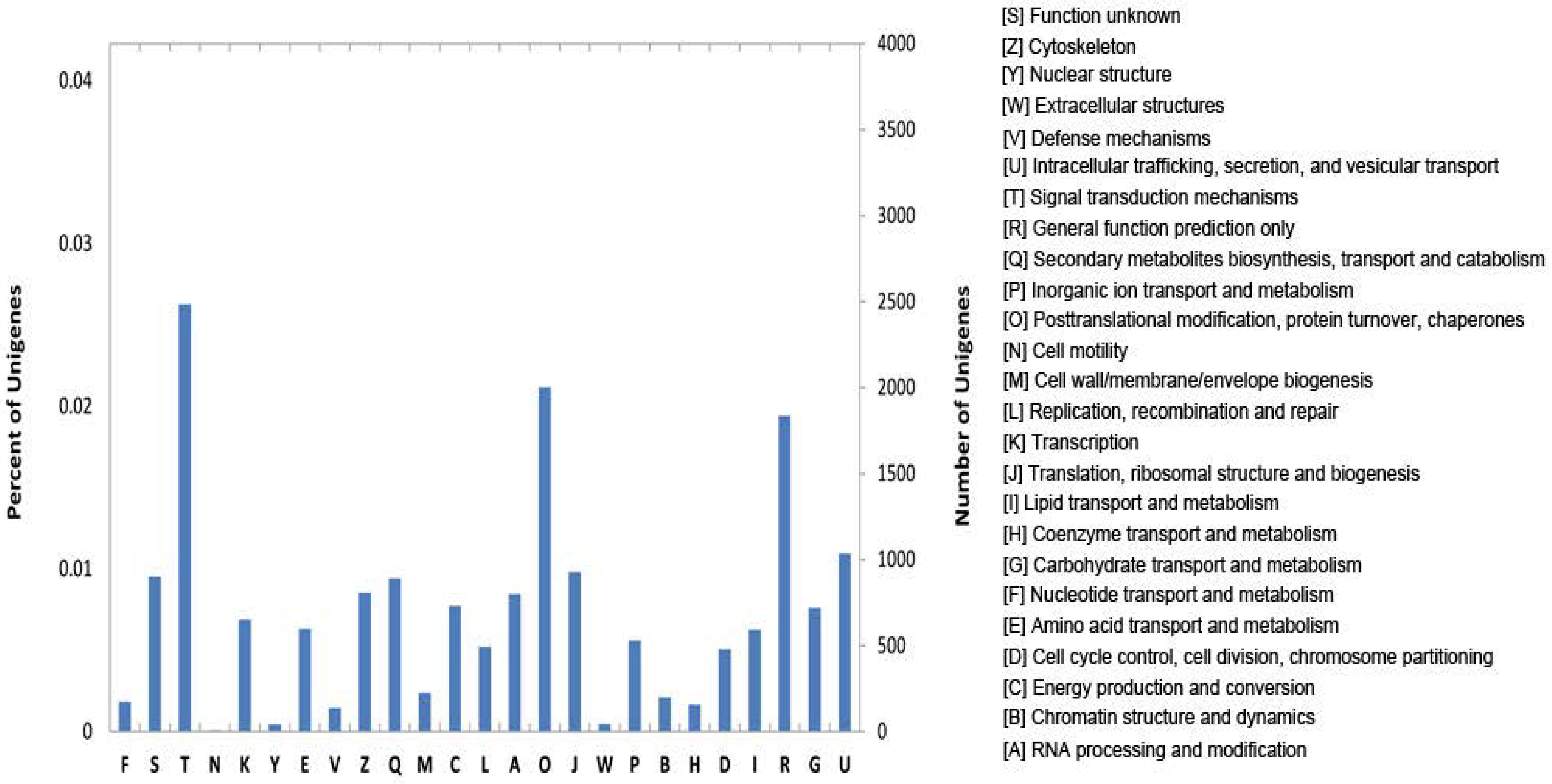

Figure S1 COGs functional categories.

Table S1 Summary of sequencing and assembly results.

Sample	Raw Data		Valid Data			Valid Ratio (reads)
	Read	Base	Read	Base	Average length	
0 h	61582552	6158255200	51653088	4894314337	94.75	83.88%
12 h	49440532	4944053200	41461690	3920873241	94.57	83.86%
24 h	46627782	4662778200	38831566	3666484391	94.42	83.28%
SUM	157650866	15765086600	131946344	12481671969	94.6	83.70%

Table S2 The top ten species with the greatest similarity by selecting 500,000 reads randomly from post-quality control date to blast against nucleotide database for empoison detection.

Species	Reads number
Populus trichocarpa	672570
Populus tremula x *Populus alba*	136372
Populus trichocarpa x *Populus deltoides*	12713
Populus tremula	6176
Populus tomentosa	3369
Populus tremula x *Populus tremuloides*	3333
Populus tremuloides	3301
Populus balsamifera	3056
Populus alba x *Populus tremula var. glandulosa*	2750
Picea glauca	1571

Table S3 The number of differentially expressed unigenes base on different analysis method. FC.p: fold change AB positive, log2(B/A)>1, FC.n: fold change AB negative, log2(B/A)<-1, FT.g: fisher.testAB.greater, FT.l: fisher.testAB.less, CT: chisq.test.AB.

	FC.p（up）	FC.n（down）	FT.g	FT.l	CT
12/0 h	32327	29921	1255	1648	6773
24/12 h	27888	25824	1156	774	6796

Table S4 The top ten pathways active in the transcriptome of all samples and the number of unigenes mapped to those pawthays.

Pathway	Pathway_type	Unigene_num
ko03010, Ribosome	Genetic Information Processing/Translation	610
ko04075, Plant hormone signal transduction	Environmental Information Processing/Signal Transduction	475
ko04141, Protein processing in endoplasmic reticulum	Genetic Information Processing/Folding, Sorting and Degradation	460
ko00500, Starch and sucrose metabolism	Metabolism/Carbohydrate Metabolism	441
ko03040, Spliceosome	Genetic Information Processing/Transcription	397
ko00230, Purine metabolism	Metabolism/Nucleotide Metabolism	350
ko03013, RNA transport	Genetic Information Processing/Translation	349
ko00190, Oxidative phosphorylation	Metabolism/Energy Metabolism	349
ko04626, Plant-pathogen interaction	Organismal Systems/Environmental Adaptation	334
ko00240, Pyrimidine metabolism	Metabolism/Nucleotide Metabolism	281

Table S5 Primers utilized in RT-qPCR.

gene name	transcript ID	primers	Oligonucleotide	Fragment length(bp)	Amplification efficiency (%)	Homologs GenBank ID
Flavine monooxygenase	comp37974_c0_seq1	forward	5'-GTTGGCCTCTGAATCCCATATTTG-3'	190	99.56	EEE93669.1
		reverse	5'-ATTTGCTCTATTGGGGTGCTCATA-3'			
CytochromeP450 83B1	comp97971_c0_seq5	forward	5'-TCAGCTAAAATGGCCAGAGAGATT-3'	206	100.32	EEE79998.1
		reverse	5'-TCTCTAATGGGACGGAAACTTTGT-3'			
GA20-oxidase	comp78544_c0_seq1	forward	5'-GAAAGAGTGTTCATGGCTTCACAA-3'	176	102.51	EEE98398.1
		reverse	5'-TTCACTGGAAGATTCTCCTCCAAG-3'			
ACC synthase	comp508576_c0_seq1	forward	5'-TACGTTTCATTGCAACTTTGAGAG-3'	173	101.22	EEE81653.1
		reverse	5'-TCTCTTAAGCTCTCAGACCTTTGA-3'			
ACC oxidase	comp78273_c0_seq1	forward	5'-AGGCTGTAGAATCCACAATCAACT-3'	222	100.36	EEF10883.1
		reverse	5'-TGATCACATGAACTACGCACTACA-3'			
Zeaxanthin epoxidase	comp99936_c0_seq1	forward	5'-AATGCTTTGTTTCCATGCTCTCTC-3'	235	98.68	EEE90589.1
		reverse	5'-TGAGGGTTGGTGTGATAATGTGAT-3'			
9-cis-epoxycarotenoid dioxygenase	comp89797_c0_seq1	forward	5'-CGAGTTGTCTGAAATCCGTTTGAA-3'	208	100.52	EEF44272.1
		reverse	5'-AAATTTTGTCACCTCGTTCGTCTC-3'			
CytochromeP450 90B1	comp72896_c0_seq2	forward	5'-GAAGTTCTGAACAAGGTGGTGAAG-3'	189	103.45	EEE99528.1
		reverse	5'-GTACTTTTCTGGCTTTGAGTGGAC-3'			
CytochromeP450 90D1	comp82793_c0_seq2	forward	5'-TTCACCAGGATCCAAGCTAATCAA-3'	218	100.08	EEE78017.1
		reverse	5'-TCATCAATGGGAGCTTACAAAGGA-3'			
Allene oxide synthase	comp96159_c0_seq1	forward	5'-TCATTCCACCAAACGAGTTAAAGC-3'	219	100.84	EEE81726.1
		reverse	5'-TCCTTTCTCTCGGTCTTCCAAAAT-3'			
Jasmonate O-methyltransferase	comp79559_c0_seq1	forward	5'-TGGAGCTTTTGAAGATGGTGGATA-3'	187	101.09	EEE91270.1
		reverse	5'-TCTAGTCACGGAAACAGTCACATT-3'			
Phenylalanine amomonia lyase	comp56977_c0_seq1	forward	5'-TATTCCTCGACACGTCAATCAAAG-3'	194	100.92	EEE91436.1
		reverse	5'-CGCCTATGTTAAAGAAGCTCAGAA-3'			
Ornithine decarboxylase	comp12331_c0_seq1	forward	5'-GCCACCAACATTATAGGAAAACGT-3'	223	99.38	EEE94752.1

		reverse	5'-TTAAAACCGTGTCAAGAGCATCAC-3'			
Glutamate decarboxylase	comp57791_c0_seq1	forward	5'-TGACAAGACGCTCAACATTAGAGA-3'	204	98.92	EEE74571.1
		reverse	5'-TTGGCTAATGAAGAGAAGGAAGCT-3'			
GABA Transaminase	comp95593_c0_seq9	forward	5'-TAAGTTGCTGGTGGAGGTATCAC-3'	206	100.46	EEE91436.1
		reverse	5'-GATCTTCCTGCACCATTTGTACG-3'			
Stigma-specific protein 1	comp47426_c0_seq1	forward	5'-ACTGGAGTGAAGGAAACAGTAATT-3'	156	102.29	EEE70518.1
		reverse	5'-AAAGTGTGTTGATGTTCGAAACGA-3'			
Calmodulin	comp79708_c0_seq2	forward	5'-GGGACAATAGAGTTTGCTGAGTTC-3'	226	101.56	EEF00832.1
		reverse	5'-CTTGACCATCACCATCCAAATCAG-3'			
Pectin methylesterase	comp73257_c0_seq1	forward	5'-ATATTCACCTTCCGTTCACAATGC-3'	200	100.43	EEE98605.1
		reverse	5'-AGGTATCCTTGCTTAGTTTCGTGA-3'			
Pectin methylesterase	comp452695_c0_seq1	forward	5'-GCCCAGTTAACCCTCTTTGAAATC-3'	175	100.69	EEF03906.1
		reverse	5'-GAAGGCCATGGAGAGGTTATTCTA-3'			
Pectin methylesterase	comp60715_c0_seq1	forward	5'-CGCTCATCATGGTCGATATAGTGA-3'	196	103.94	EEF00692.1
		reverse	5'-CCATCTTCAGCAAATTTAGGGTCG-3'			
Solute carrier family 25. member 39/40	comp100486_c0_seq24	forward	5'-GACTTTAAAAGCATTGGGGTCCTC-3'	184	103.57	EEF12213.1
		reverse	5'-CCTCTCCCATGTCAGAAGTACAAA-3'			
Myb proto-oncogene protein	comp94912_c0_seq1	forward	5'-ATGAGACTAACAGAAGGGGAGAGA-3'	208	102.35	EEE89502.1
		reverse	5'-GTGCTATCAAACTCCAATTCCTCG-3'			
Pectate lyase:	comp82692_c0_seq3	forward	5'-CCAGAAGATGCAACCGATGATTTT-3'	197	100.98	EEF03968.1
		reverse	5'-TTTCCTTGCTTCAGGTTCCTTCTA-3'			
Cellulose synthase:	comp627632_c0_seq1	forward	5'-AAATCGAGGTGAATGGGTAGATGC-3'	173	100.42	ERP62671.1
		reverse	5'-TGGCTCAGCTCCTATTAACCTTAC-3'			
Actin	comp96615_c0_seq1	forward	5'-GCCATCTCTCATCGGAATGGAA-3'	171	101.84	EEF11760.1
		reverse	5'-AGGGCAGTGATTTCCTTGCTCA-3'			
Ubiquitin	comp68355_c0_seq2	forward	5'-TGAGGCTTAGGGGAGGAACT-3'	195	100.96	EEE93721.1
		reverse	5'-TGTAGTCGCGAGCTGTCTTG-3'			
